# A Rare Case of Huge Schistosomiasis-Associated Cecal Polyp Mimicking Colon Carcinoma

**DOI:** 10.7759/cureus.37718

**Published:** 2023-04-17

**Authors:** Ahmed H Abdelfattah, Mubarak Ali, Ahmed A Abd El Fattah, Mostafa Ghazy, Ahmed E Eladl, Ahmed N Elkot

**Affiliations:** 1 Internal Medicine, University of Kentucky College of Medicine, Lexington, USA; 2 Internal Medicine, Mansoura International Hospital, Mansoura, EGY; 3 Gastroenterology and Hepatology, Specialized Internal Medicine Hospital, Mansoura University, Mansoura, EGY; 4 Department of Pathology, Faculty of Medicine, Mansoura University, Mansoura, EGY; 5 Internal Medicine Department/Gastroenterology and Hepatology Unit, Specialized Internal Medicine Hospital, Mansoura University, Mansoura, EGY

**Keywords:** schistomiasis polyp, intestinal bilharziasis, bilharzial polyp, colorectal cancer, cecal polyp, intestinal schistosomiasis, schistosomiasis

## Abstract

Schistosomiasis is a parasitic infection caused by Schistosoma species, commonly found in tropical and subtropical regions. It affects millions of people worldwide and can lead to different clinical presentations like abdominal pain, weight loss, anemia, and chronic colonic schistosomiasis. In rare cases, chronic infection can result in the development of polyps, which can mimic colon carcinoma, posing a diagnostic challenge. Here, we present a rare case of a huge Schistosomiasis-associated cecal polyp in a patient initially suspected to have colon cancer. The patient's clinical history and the histopathological analysis confirmed the diagnosis, emphasizing the importance of considering parasitic infections in the differential diagnosis of gastrointestinal polyps in Schistosomiasis-endemic areas. This case report highlights the need for increased awareness among healthcare professionals of the potential for Schistosomiasis-associated polyps and the importance of multidisciplinary management in such cases.

## Introduction

Schistosomiasis, a tropical parasitic infection caused by blood flukes of the Schistosoma genus, is the world's third-most prevalent devastating tropical disease. The disease is endemic in 74 countries across the Middle East, Africa, South America, Asia, and the Caribbean, infecting approximately 250 million people and rendering about 700 million people at risk of infection [1]. To date, seven species have been identified to infect humans [2]. Intestinal schistosomiasis, caused primarily by *Schistosoma mansoni* in the Middle East, is a parasitic infection characterized by the migration of larvae against the portal circulation and subsequent deposition of eggs in the walls and mesenteries of the large intestine and rectum. Some of these eggs dig their way through the intestinal wall leading to minute ulcers that cause bleeding. However, the eggs that remain trapped in the intestinal wall evoke a granulomatous immune response. Over time, the accumulation of these granulomas can lead to the formation of polyps which may protrude into the intestinal lumen and can, in rare cases, be mistaken for malignant polyps.

Schistosomiasis infection has rarely been reported to cause huge cecal polyps, and a massive polyp presented as the sole manifestation of intestinal bilharziasis is even less frequently encountered. This presentation can be easily confused for a malignant polyp because of the overlap in clinical manifestations and endoscopic appearance of both diseases [3,4].

Here we report a case of a huge bilharzial polyp originating from the ileocecal junction which caused a partial obstruction of the cecal lumen as a manifestation of bilharzial colitis.

## Case presentation

We present a case of a 52-year-old male Egyptian farmer who presented to our clinic with a primary complaint of right lower quadrant abdominal pain, constipation, and vomiting. The pain was colicky in nature, gradual in onset, progressively worsening over one and a half months, and associated with weight loss. It did not increase or decrease with food intake. He did not have a significant past medical history. He lived in a village and was frequently exposed to unhealthy water. He was from low socio-economic status and had no family history of bilharzial disease or cancer. Social history was considered irrelevant. Physical examination showed that his vials were stable, with general physical examination positive for pallor in the conjunctiva and palms. Abdominal examination showed a lax abdomen with some tenderness in the right lower quadrant. No mass was palpated. The digital rectal exam (DRE) was normal. His laboratory findings were significant for anemia, and the eosinophils were on the upper normal limit (Table 1).

**Table 1:** Laboratory findings. * Grams per deciliter ** Milligrams per decilitre

Complete Blood Count with Differential	Results	Normal Range
Hemoglobin	10.5	13.1-17.2 g/dl *
RBC count:	3.9 × 10^6^	cells/µL
Hematocrit	38.9	39 – 50%
Mean corpuscular volume	99.7	80 – 100fL/cell
Mean corpuscular hemoglobin	26.9	27 – 34 pg/cell
Mean corpuscular hemoglobin concentration	27	32 – 37 g/dl
White blood cell	3.00	4.5 – 11.0 × 10^3^/mm^3^
Neutrophils	60	35.0 – 80.0 %
Lymphocytes:	35	18 – 44 %
Monocytes:	2	0 – 10 %
Eosinophils:	3	0 – 3 %
Platelets	90	150 – 400 × 10^3^/mm^3^
Creatinine	0.8	0.6 – 1.2 mg/dl **
Blood Urea Nitrogen	28	15 – 45 mg/dl **
Albumin	3.6	3/8 – 5/2 g/dl
INR	1.2	1.0 – 1.3
Hep B sAg	-ve	
Hep C IgG	-ve	
Random Blood glucose	98	Up to 200 mg/dl

**Figure 1:**
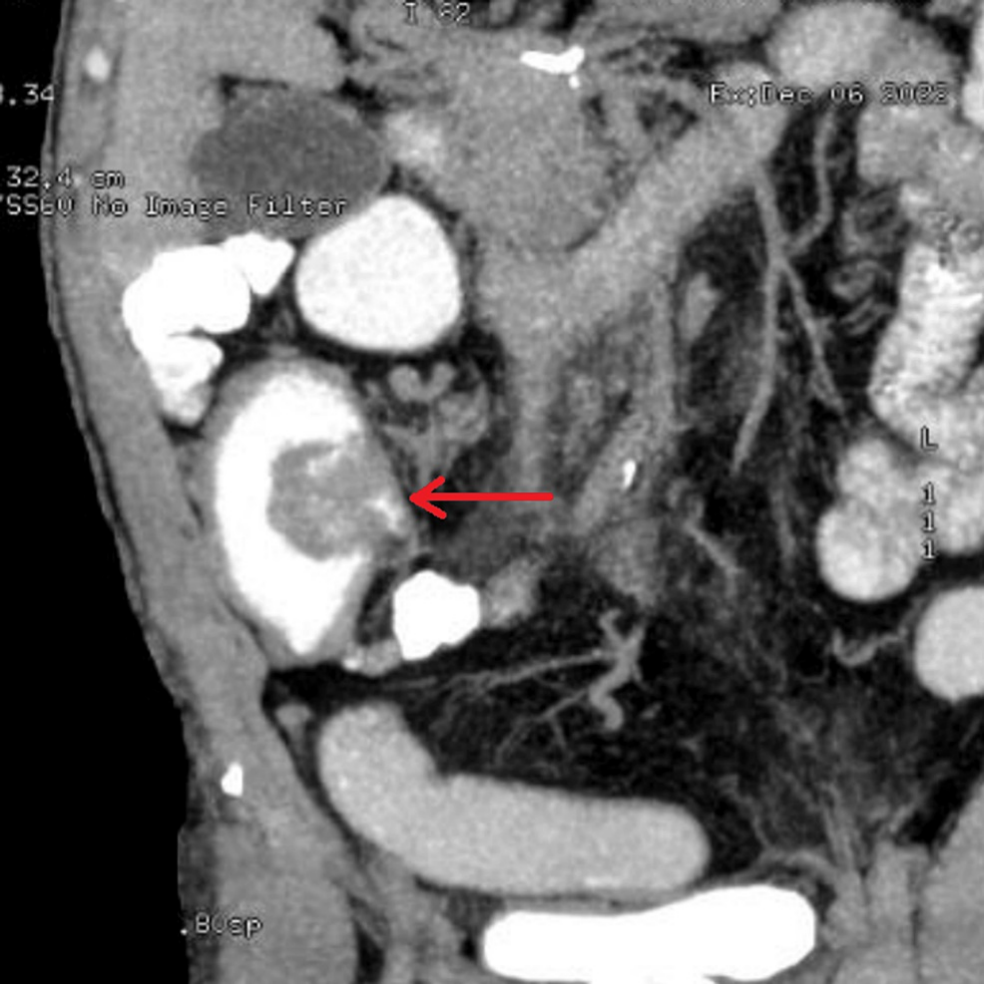
CT abdomen and pelvis with a red arrow pointing to a well-defined homogenously enhanced soft tissue mass at the ileocecal junction

CT scan of the abdomen and pelvis with oral and IV contrast showed a well-defined, homogenously enhanced soft tissue mass at the ileocecal junction measuring about 5×5×4 cm which was protruding into the cecum and thereby markedly attenuating the lumen. In addition, moderately dilated distal ileal loops with smudging of surrounding fat planes were noted (Figure 1).

**Figure 2:**
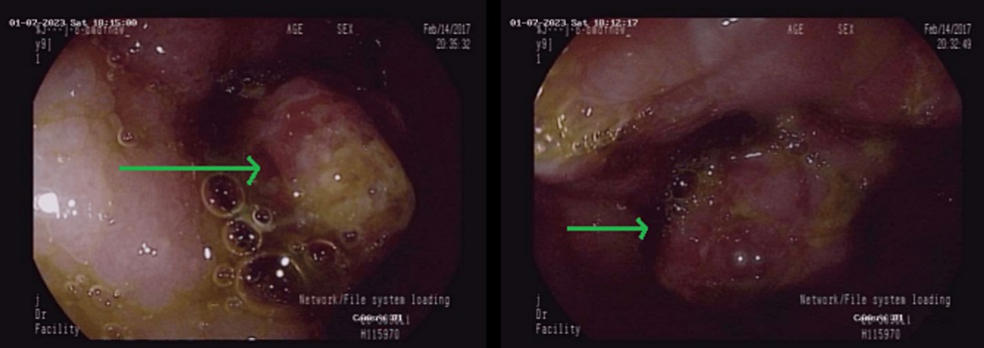
Colonoscopy shows a large cauliflower polypoid mass protruding from the ileocecal valve (green arrows)

He was then referred for an elective colonoscopy, revealing a large cauliflower polypoid mass protruding from the ileocecal valve and markedly obscuring the lumen. The size of the polyp was measured to be 5 × 5 cm (Figure 2 and Video 1). 

**Video 1:** Schistosomiasis-associated cecal polyp mimicking colon carcinoma

**Figure 3:**
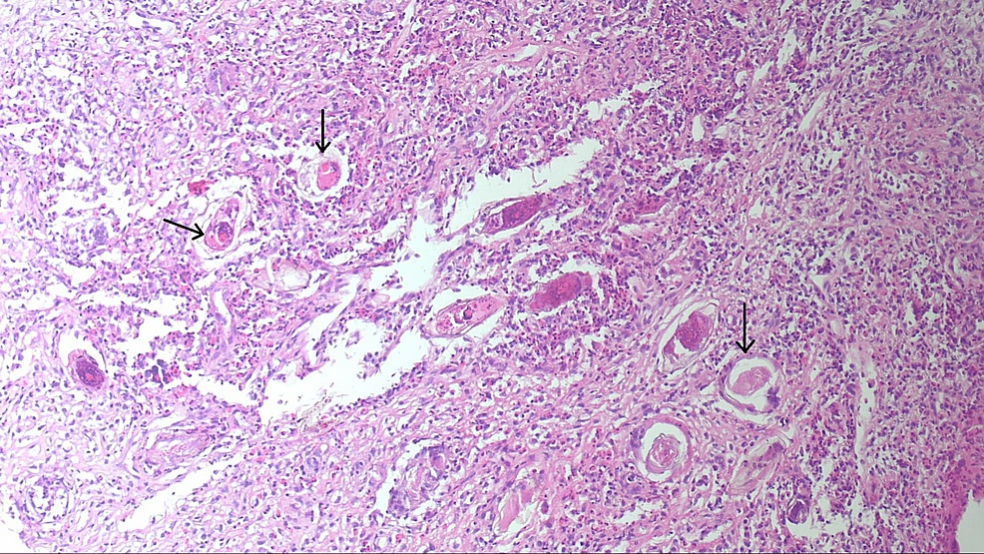
Histopathological slide with multiple black arrows showing the bilharzial granulomas

Multiple biopsies were taken for histopathology, which showed multiple viable bilharzial ova with related granuloma in the submucosa (Figure 3).

Since the polyp was huge and the patient was at risk of developing complications such as bowel obstruction, he was referred to the surgical team. The patient underwent a right hemicolectomy with ileotransverse anastomosis. The microscopic examination of the specimen showed bilharzial granulomas formed of numerous viable bilharzial ova surrounded by epithelioid cells, giant cells, and excess eosinophils. No atypia or malignancy was noted, confirming the diagnosis of bilharzial colitis.

## Discussion

Schistosomiasis is the third-most devastating tropical disease across the globe caused by a blood fluke of the genus Schistosoma (S). To date, seven species have been described to cause infections in humans, of which *S. hematobium, S. mansoni, *and *S. japonicum *are more prevalent, with the disease burden estimated to be between 24-29 million disability-adjusted life years [2]. The most common culprits of intestinal schistosomiasis are *S. mansoni *and *S. japonicum* [5]. These organisms travel against the portal circulation to reach the gastrointestinal tract and lay their eggs in the walls and mesenteries of the large intestine and rectum. Some of those eggs evoke a granulomatous response, leading to granuloma formations, fibrosis, obstruction, and sometimes polyps formation [6].

Bilharzial polyp formation starts with a submucosal granuloma elevating the mucosa above it, and the polyp grows in size with the accumulation of more granulomas. As the polyp grows, the mucosa surrounding and overlying it proliferates, and the stalk begins to form. A layer of functional mucosa covering a core of connective tissue with a blood supply makes up the stalk. The body of the polyp is made up of the same epithelium as the small bilharzial granuloma and comprises many goblet cells that secrete a large amount of mucus. Because of its fragility and high vascularity, it bleeds easily when touching or passing stools [7].

Bilharzial polyps can be encountered anywhere in the colon, with a preference for the recto-sigmoid region [8,9]. The polyp in our patient was found at the ileocecal junction, which is a rare manifestation, and was obstructing the lumen to such an extent that the distal ileum appeared dilated on the CT scan. Al-Antably et al. investigated socio-demographic risk factors of *Schistosoma mansoni* in patients with gastrointestinal symptoms in Egypt. They found that 72.6% of the study population with high schistosomiasis titers had positive contact with canal water. Additionally, the male gender was also found to be a risk factor [10]. Our patient is a male farmer who lives in Egypt and has a history of contact with unclean canal water, putting him at risk of contracting the parasite.

Patients with bilharzial polyps can present with vague and non-specific symptoms such as abdominal pain, nausea, vomiting, bleeding per rectum, diarrhea, or constipation which can occur in a multitude of gastrointestinal (GI) pathologies [3,6]. Issa et al. reported a right-sided bilharzial polyp as a cause of long-standing abdominal pain in a 20-year-old Ethiopian woman [8]. Bilharzial polyps are also documented to cause appendicitis [11]. Some other rare manifestations include intestinal intussusception, GI obstruction, and colon cancer [8].

The diagnosis of intestinal schistosomiasis can be made by egg detection from stool samples or endoscopy biopsy. Urine and serum serological antibody detection can be beneficial to rule out infection in epidemic regions but its sensitivity and specificity can sometimes be problematic in non-epidemic regions [12]. As the bilharzial polyps look the same as any other polyp, the CT and endoscopic findings are often nonconclusive and nonspecific. Historically, a rectal biopsy of the lesion and examination under the microscope has been used as an effective modality of diagnosis. 

The treatment of suspected bilharzial polyps involves praziquantel, with recommended doses of 40mg/kg in one or two doses for S.* hematobium*, S.* mansoni*, and S. *intercalatum* and 60mg/kg in two or three doses for S. *japonicum* and S. *mekongi*. ​​​​​​Medical treatment alone is effective against the earlier stage of intestinal bilharziasis. Endoscopic removal is considered the gold standard for small polyps, but in cases of large polyps, surgical intervention may be required. Due to the rarity of this condition, there is no specific guidance for the management of partial colonic obstruction [13] In our case, the patient had a large risky polyp in the cecum to the extent of incomplete bowel obstruction, so the patient was referred for a right hemicolectomy to remove the polyp. No effort for endoscopic removal was made due to the risk of complications.

We recommend that physicians should not be dissuaded from considering a diagnosis of bilharzial polyp merely because of the size of the polyp, as it can manifest solely as a giant polyp. We believe the physicians practicing in Schistosoma-prevalent areas should have a low threshold of suspicion for bilharzial polyps. Notably, bilharzial polyps can be formed anywhere in the large intestine, including the ileocecal junction. We believe that bilharzial polyp should be on the differential in the workup of a colorectal polyp or carcinoma patient. Risk factors, like traveling to any endemic area should be explored, as even a single contact with the contaminated water may lead to a huge bilharzial polyp mimicking colorectal cancer (CRC).

## Conclusions

Schistosomiasis is a significant health concern in endemic regions across the globe. A single massive polyp in the cecum may be the sole manifestation of intestinal schistosomiasis and be considered in the workup of any cecal or colonic polyp. Sometimes the polyp can be huge enough to cause partial intestinal obstruction. Physicians working in Schistosoma-endemic areas should have a low threshold of suspicion for bilharzial polyp and order diagnostic tests to rule it out to avoid unnecessary surgeries. Praziquantel is an effective modality of treatment for all Schistosoma species.
